# Lexicon for classifying ear-canal shapes

**DOI:** 10.1038/s41598-023-38570-3

**Published:** 2023-07-22

**Authors:** J. C. Martinez, Goh Zhi Hwee, Luis Yap, Kenneth Wei De Chua, Savitha Kamath, Conrad Kang Rui Chung, Wendy Yu Bing Teo, Charmaine Kai Ling Tan, Stylianos Dritsas, Robert E. Simpson

**Affiliations:** 1 Singapore University of Technology and Design (SUTD), 8 Somapah Road, Singapore, 487372 Singapore; 2 University of Sydney, Sydney, NSW 2006 Australia; 3 Department of Otorhinolaryngology-Head and Neck Surgery, Changi General Hospital, Singapore, Singapore; 4 School of Engineering, University of Birmingham, Birmingham, B15 2TT UK

**Keywords:** Ageing, Physiology, Anatomy, Mathematics and computing

## Abstract

The ear canal is usually described as an S-shaped funnel. In attempting to classify ear-canal shapes obtained from point clouds digitized from molds of 300 ears, the problem of designing criteria for distinguishing and organizing the canal shapes arose. In this work, we extracted features inspired by the S-shape characteristic (critical point, maximum, minimum, twist, writhe, translation, rotation) and, through them, introduced 14 types of ear-canal shapes. This classification allowed comparison of ears within a type and of ears between different types. It expanded our range of descriptors of canal shapes and unlocked perspectives for applications.

## Introduction

The ear canal connects the pinna, which is on the outside of the ear, to the ear drum and measures approximately 2.5 cm long, varying with gender, age and ethnicity^1^. An accurate description of the ear canal is scarce in the literature and it is surprising that few have studied the normal ear canal in detail. It is more common to find descriptions of pathological ear types than of normal ones^2–4^. A good understanding of the ear canal shape has implications downstream on hearing-aid amplification, as it allows for accurate positioning of the receiver and a snug fit of the ear mold^5^. Conventionally, ear impressions are taken by audiologists to reconstruct the shape of the canal and facilitate ear-mold making. Although this may seem like the most accurate representation of the canal shape, patients still evidently complain about ill-fitted hearing aids that require modification and poor fits seem to be one of the barriers to hearing-aid adoption^6^. Indeed, improving the comfort for hearing-aid users is a growing problem^7^. It is estimated that by 2050, approximately 900 million people worldwide will suffer from hearing loss^8^. However, hearing aid uptake remains disproportionate to the estimated number of people who need them. Amongst the many reasons for hearing aid acquisition, the fit and comfort from ear molds may be vital as patients are expected to wear their hearing aids for extended periods of time^9^.

The ear canal shape is complex, and for hearing aids to be fitted properly and then adopted broadly, techniques must be developed to accurately produce custom hearing aids. Three-dimensional (3D) scanning of the ear impression provides high quality images for a good estimation of the canal shape. The canal itself is not straight; it bends and twists as it advances toward the ear drum. One of the most common descriptions of the ear canal is that it is “*S shaped”*^10^. Clearly, the S-shape description is a projection onto a plane; in reality the S-shape is not flat but also has a pitch in 3D space. If one imagines cutting thin transverse slices of the canal along its length, one finds the slices to be elliptic cross sections with major axis length varying unevenly within the canal between 10 and 17 mm and minor axis length between 3 and 6 mm. When comparing the slices, they appear to rotate and tilt as one looks at those further ahead, reflecting the bends and twists of the canal itself. Perhaps a more accurate description of the ear canal is that it “writhes” in space. A detailed knowledge of the shape and dimensions of the canal is essential if hearing aid receivers and ear molds are to fit comfortably in the canal.

Herein, we propose a classification system for ear canal shapes. To our knowledge no such attempt has yet appeared although the problem has been appreciated for some time^11,12^. Perhaps the most immediate idea that leaps to mind when attempting to classify ears is the topological one, which relies on diffeomorphism, i.e., shapes like the canal are thought of as amorphous rubber tubes and they are distinct if they cannot be deformed one to another without crumpling, cutting or folding, while stretching and squeezing, rotations and translations are acceptable manoeuvres^13^. In this respect all normal ears are diffeomorphic to each other so distinctions cannot be made, at least for adults^14^. We start with the accepted observation that the ear canal is S-shaped. We broaden this insight by introducing the concepts of critical points, writhe, and twist, rendering them more precise for our objective. We find that these concepts provide tools for classifying and comparing canals. Furthermore, the descriptors proposed here, provide quantitative measures for comparing ear canals.

## Method

### Framework and shortcomings

**Figure 1:**
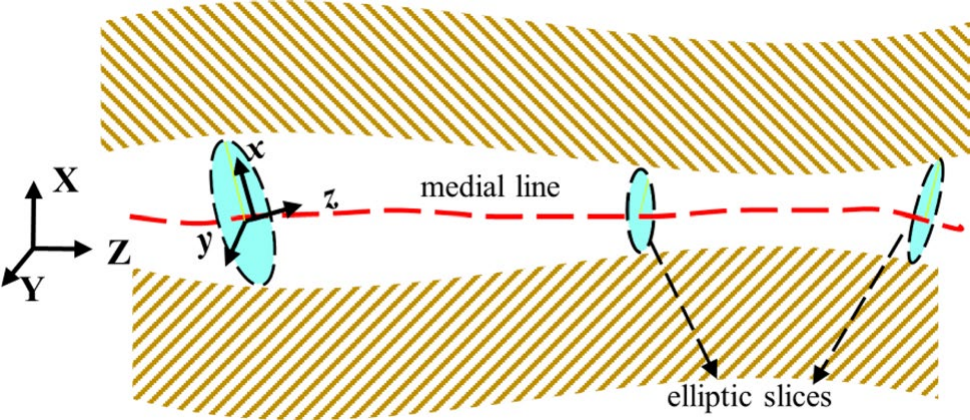
Schematic diagram of the initial elements used to describe the shape of the ear canal.

To arrive at a detailed knowledge of the ear canal, we imagine that advancing into the canal is equivalent to the motion of a reference frame (shown as *xyz* in Fig. 1), which undergoes 3D translations and rotations, as it navigates through the canal toward the ear drum. We also imagine that elliptic slices, as described above, have been made of the canal. At any instantaneous position in the canal, the mobile frame has the elliptic slice on its *xy* plane and its normal is also its z-axis. Another ‘XYZ’ reference frame is fixed outside the ear from which the position and orientation of any slice is measured. We will give more detail in subsequent paragraphs. Together with the semi-major and semi-minor axes lengths, the coordinates of the center of the ellipse (or the mobile reference frame) along with the Euler angles parametrizing its orientation are obtained. The curve joining the centers of the slices is called the *medial line*.

The current method to obtain in situ information about the shape of the canal begins with an ENT clinician injecting an impression material into the canal, which is then extracted after curing and hardening^15^. The resulting mold is then scanned to create a 3D map of the canal. After processing the scan and deciding how to incorporate the hearing aid components, a hearing aid shell is 3D printed. Here, we started with the 3D scan point cloud of the canal. For hearing aids, it is unnecessary to know the shape of the *entire* canal; it is sufficient to have information on the first half of the canal that is closest to the pinna because the hearing aid generally occupies that space. We therefore restricted our study to this half of the canal.

**Figure 2:**
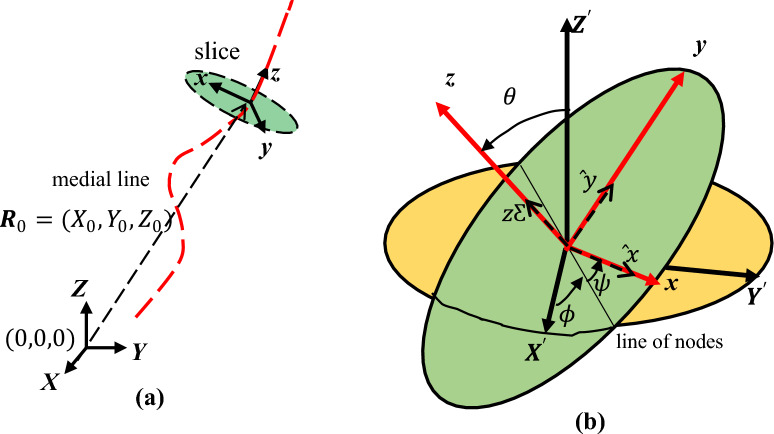
(**a**) As the mobile frame moves along the medial line to a given elliptic slice, the coordinates (*X*_0_*, Y*_0_*, Z*_0_) of its center are measured from the stationary XYZ reference frame outside the ear. Note that the origin of the *XYZ* frame is not generally at the entrance of the canal. (**b**) The Euler angles of a slice are determined from the frame ${X}{^\prime}{Y}{^\prime}{Z}{^\prime},$ which is the parallel transport of the XYZ reference frame to the center of the slice.

The point cloud is an ensemble of data points (roughly 70,000) giving the coordinates of the points of the canal wall. Since the molds were obtained by different audiologist clinicians and in varying circumstances, each mold had its own characteristic features. It was not possible to standardize this step. Hence the position and orientation of the XYZ reference frame was left arbitrary, except that its XY plane was approximately flat with the pinna of the ear and its Z*-*axis pointing into the ear. See Fig. 1. This means that data points do not necessarily start at the entrance of the canal, though it is close. This fact is noted in several places in the results reported below. Each ear canal point cloud was processed, yielding data for between 12 to 36 elliptic cross sections, depending on the length of the canal cloud. The average number of cross sections was in the low 20 s. For every elliptic cross section, the data consisted of: (a) the coordinates of its center (*X*_0_*, Y*_0_*, Z*_0_), as displayed in Fig. 2a; (b) the directions of the semi-major, the semi-minor axes and the normal $\widehat{{\varvec{z}}}$**,** respectively along with the (c) lengths of these axes. See Fig. 2b. These data were obtained from the point clouds with the aid of the *Rhino* software. With these data the corresponding Euler angles ($\theta ,\phi ,\psi$) of each cross section was computed. To obtain this information we also employed another reference frame, $({X}{^\prime}{Y}{^\prime}{Z}{^\prime})$, with origin at the center of the elliptic slice and whose axes are parallel to the those of the external XYZ frame. ***R***_0_ is the vector connecting these two origins.

### Quantitative aspects

We take the following convention for the Euler angles: starting with the frame $X{^\prime}Y{^\prime}Z{^\prime}$ in Fig. 2b: (a) rotate it by an angle $\phi$ about the $Z{^\prime}$-axis (this rotation brings the axis $X{^\prime}$ to the line of nodes); (b) then rotate by an angle $\theta$ about the line of nodes (this brings axis $Z{^\prime}$ to the *z*-axis (shown in red)) and (c) finally rotate by an angle $\psi$ about the *z-*axis^16^. These rotations are not commutative. Based on Fig. 2b we have the following relations for the Euler angles

1 $$\cos \theta = \hat{\user2{z}} \cdot {\hat{\mathbf{Z}}}^{\prime } ,\cos \phi = \hat{\user2{X}}^{\prime } \cdot \left( {{\hat{\mathbf{Z}}}^{\prime } \times \hat{\user2{z}}} \right),\cos \psi = \hat{\user2{x}} \cdot \left( {{\hat{\mathbf{Z}}}^{\prime } \times \hat{\user2{z}}} \right).$$

By convention, all vectors with carets have unit length.

To obtain the equation of the elliptic-slice perimeter from the stationary *XYZ* frame, recall that the parametric equations of an ellipse with center at the origin, flat on the *XY*-plane and with major (minor) axis in the *X*-direction (*Y*-direction), is $X=a\mathrm{ cos}t,Y=b\mathrm{sin}t$, *a* and *b* being the semi-major and semi-minor axes lengths, respectively and the parameter $0\le t\le 2\pi$. The elliptic slice is obtained from this ellipse by first translating its center to the origin of the frame (this is the vector ***R***_0_ in Fig. 2a) and then performing the appropriate Euler rotations to bring the frame in coincidence with the *xyz* system of the slice (see Fig. 1). The overall rotation matrix that effects this is

2 $${\text{E}} = \left( {\begin{array}{*{20}c} {\cos \psi \cos \phi - \cos \theta \sin \phi \sin \psi } & { - \sin \psi \cos \phi - \cos \theta \sin \phi \cos \psi } & {\sin \theta \sin \phi } \\ {\cos \psi \sin \phi + \cos \theta \cos \phi \sin \psi } & { - \sin \psi \sin \phi + \cos \theta \cos \phi \cos \psi } & { - \sin \theta \cos \phi } \\ {\sin \theta \sin \psi } & {\sin \theta \cos \psi } & {\cos \theta } \\ \end{array} } \right),$$

expressed in terms of the Euler angles. The parametric equation for the slice perimeter is then

3 $$\left(\begin{array}{c}X(t)\\ Y(t)\\ Z(t)\end{array}\right)={{\varvec{R}}}_{0}+\mathrm{\rm E}\left(\begin{array}{c}a\mathrm{cos}t\\ b\mathrm{sin}t\\ 0\end{array}\right)$$

Two other quantities are useful to calculate. The ‘twisting rate’ as seen at the slice frame is^17^

4 $${\omega }_{Z}=\frac{d\phi }{dZ}cos\theta +\frac{d\psi }{dZ}$$

Here, *Z* represents z-coordinate as seen from the stationary XYZ reference frame. From this we obtain total twist (or twist for short)

5 $$\mathrm{Totaltwist}=\frac{1}{2\pi }{\int }_{{Z}_{1}}^{{Z}_{2}}\left(\frac{d\phi }{dZ}\mathrm{cos}\theta +\frac{d\psi }{dZ}\right)dZ=\frac{1}{2\pi }{\left.\psi \right|}_{{Z}_{1}}^{{Z}_{2}}+\frac{1}{2\pi }{\int }_{{Z}_{1}}^{{Z}_{2}}\frac{d\phi }{dZ}\mathrm{cos}\theta dZ$$

*Z*_1_ and *Z*_2_ are the *Z*-coordinates of the first and last elliptic slices, respectively. The last integral is computed numerically. The calculations in the paper were carried out using the *Mathematica* software. In the context of the ear canal, the twist represents the total turn undergone by the major axis in the course of arriving at its final slice position. Stated differently the twist is the total turn of the mobile frame about its normal as it follows the stack of elliptic slices. A total twist of unity means one full turn.

The twisting rate contains both $\frac{d\phi }{dZ}$ and $\frac{d\psi }{dZ}$. This observation will be important later in our development because the twisting rate consolidates the turning due separately to $\phi$ and $\uppsi$. It represents a kind of overall twisting as one advances into the ear canal.

The other quantity of interest is the ‘writhe’^18^:

6 $$\mathrm{Wr}=\frac{1}{2\pi }{\int }_{{Z}_{1}}^{{Z}_{2}}(1-\left|\mathrm{cos}\theta \right|)\frac{d\phi }{dZ}dZ$$

For a curve in 3D for which *ds*/*dZ* > 0 (i.e. rate of change of arclength *s* with *Z*), its local tangent traces a curve in the northern hemisphere of the trantrix sphere. The trantrix sphere is the map of points of a curve to a unit sphere through the tangent vector at each point of the curve. The writhe is the area bounded by the tantrix curve and the arcs joining the ends to the North Pole. A technical aside: writhe is usually computed for a strip of nonzero width. For the ear we can think of the curves joining the ends (front end and back end) of the major axes as the edges of this strip.

The writhe is a measure of the ‘3D-ness’ of the ear canal. If the ear canal is a straight cylinder, then it resembles a long pole which could be regarded as a 1D object. But if the cylinder not only twists but even appears to bend into itself, then it can no longer be viewed as a linear or planar object: it is a truly 3D object. ‘Bending into itself’ is sometimes referred to as contortion^19^. Note that twist and writhe are distinct since the long 1D pole we have just referred to may have twist but with no writhe.

**Figure 3:**
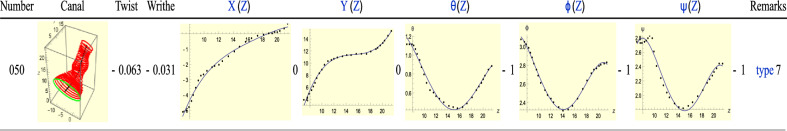
Data on ear 050 obtained via the above analysis.

It is informative to examine the typical descriptors from this analysis. Figure 3 displays a sample result for a single ear canal. The first column gives the serial number identifying the ear, while the second displays the stack of ellipses, 29 in all for this particular ear. The first ellipse at the bottom and in green is the first cross section and marks the entrance to the canal (or close to it). We see the canal contracting and expanding as it continues toward the ear drum. All lengths for all similar graphs are in mm. The third column gives the total twist: a negative sign indicates an anticlockwise turn. It is rather small compared with unity, implying little twisting. The fourth column gives the writhe which is also small. With few exceptions the writhe in all the ears we examined was small. The fifth column gives *X*(*Z*) which is the *X*-component of the medial line, with *Z* giving the distance in mm in the *Z*-direction. Along with the data points we also obtained a quartic polynomial fit of it, which is the black curve passing through the points or close by them. Similar remarks hold for the *Y*(*Z*) column. Analogous to *X*(*Z*) and *Y*(*Z*), the seventh to the ninths columns give the Euler angles of the elliptic slices. In all these five graphs the horizontal axis is the *Z*-coordinate. The graphs X(Z) and *Y*(*Z*) may have critical points, i.e., points at which the graph has a vanishing gradient. A maximum is assigned a value of + 1 while a minimum a value of – 1. These are the indices of the *critical* points.

The analysis of critical points on surfaces originated from the work of M. Morse in the 1930s^20^. Today, Morse theory is a flourishing sub-discipline of differential topology and has found application in both pure and applied mathematics^21,22^. Here, we use these topological critical points to group and categorize the different types of ears seen in the 300 sample dataset. The integers to the right of any of the five graphs in Fig. 3 are the *sums* of the indices. If there is a maximum and minimum in the *same* graph then this number is 1 − 1 = 0; if there is a third maximum in that graph then the number is now 1 − 1 + 1 =  + 1. In this example case there is no maximum/minimum for *X*(*Z*) and *Y*(Z) so the entries are both 0. We will *also* use the term index to refer to these sums. Since there is only one minimum each for the rotational graphs of Fig. 3, then each rotational graph has an index of − 1. To count these indices we sometimes relied on the data points more than on the polynomial fits to the graphs, when we had reason to doubt the reliability of the fits. The last column is for short remarks. We refer to *X, Y, Z*, $\theta ,\phi ,\psi$ as *degrees of freedom* (d.o.f).

### Procedure

We examined 300 ears and first classified them as right and left. Then for each group, we further subdivided them into different types by comparing the sum of indices of the *X*(*Z*) and *Y*(*Z*) plots with the corresponding sums for the $\theta \left(Z\right),\phi \left(Z\right),\psi \left(Z\right)$ plots. We refer to these two sets as ‘translational’ *T* and ‘rotational sums’ *R*, respectively and define them as

7 $$\begin{aligned} & T = {\text{index}}\,X\left( Z \right) + {\text{index}}\,Y\left( Z \right) \\ & R \equiv {\text{index}}\,\theta \left( Z \right) + {\text{index}}\,\phi \left( Z \right) + {\text{index}}\,\psi \left( Z \right) \\ \end{aligned}$$

By comparing *T* and *R* we are looking for some possible correlation between the “translational” aspects with the “rotational” aspects of the ear canal vis a vis our model as outlined above.

To draw the slices, we made use of the axes of the ellipses, their directions and lengths. Generally, the axes’ lengths are larger at the entrance, decreasing inside the ear and could increase somewhat toward the end. We did not make use of the information on lengths of the axes in our analysis because the change in the lengths was slower than those of the translational and rotation degrees of freedom.

**Figure 4:**
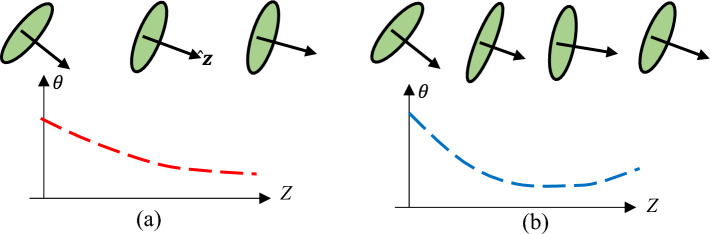
Schematic trends of the orientation of the normal (arrow) to the slices as one goes into the canal (increasing *Z*). (**a**) On the left, $\theta$ decreases monotonically, (**b**) while on the right, it reaches a minimum and increasing further on. Note that due to the arbitrariness of the orientation of the *XYZ* axes outside the ear, the minimum of the graphs need *not* be at $\theta =0$. In fact, it generally is not.

A general feature occurring practically in all the ears is for the $\theta$-plot to start with some value at the entrance of the canal and gradually decrease monotonically inside the canal as shown in Fig. 4a. In some cases, as in Fig. 4b, there is a minimum followed by an increase toward the end of the canal. Because $\theta$-motion is a tilting of the elliptic axis, unlike $\phi$ and $\psi$ motions, which are rotations *about* an axis, the need for coordination between the translations and rotations is less strongly observed for $\theta$-motion.

We had mentioned above that our study focused only on the first half of the ear canal. We note that the descriptors for two halves of the canal vary as continuous functions, and therefore it is likely that their shapes are related Indeed, the present study suggests that the outer-ear shape is correlated to the *entire* ear canal anatomy. Our future work involves exploiting such correlations using a neural network to infer ear canal shapes from limited information gained from the *outer* ear.

## Results and observations

The results below are presented such that each set of figures is accompanied by a description. This is because of the fairly large number of ears displayed. The description is not exhaustive; it simply shows salient features that reveal the ear shapes and how comparisons can be made among them. We first look at the left ears and continue with the right ears.

**Figure 5:**
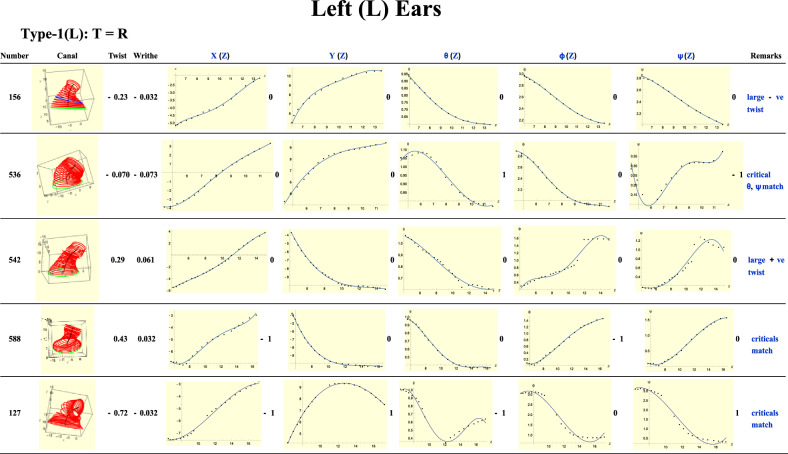
A sample of Type-1 (left) ears. All lengths for graphs are in mm.

Type-1 are those ear for which *T* = *R*. A sample of graphs is shown in Fig. 5. Comparing the first two, ears 156 and 536, we observe that both have similar translational and rotational graphs, differing notably in their $\psi$-graphs. *T* = 0 for both ears. For ear 156, all rotations are monotonic (there are no maxima or minima) and each has a zero index, hence *R* = 0 for this ear. The indices of the critical points for the $\theta$ and $\psi$ graphs of 536 are 1 and -1 (there is a maximum and a minimum) which ‘cancel,’ so *R* = 0. In other words, introducing critical points to $\theta$ and $\psi$ requires one of them to be a maximum and the other to be a minimum because *T* = *R* for Type-1 ears. Observe also the large negative twist in ear 156 and small twist in ear 536. In the latter, the rotations $\phi$ and $\psi$, being in *opposite* senses, cancel each other leading to the small twist. For ear 156 these rotations are in the *same* sense and therefore add up to the large twist. We will see this interplay in many of the ears.

**Figure 6:**
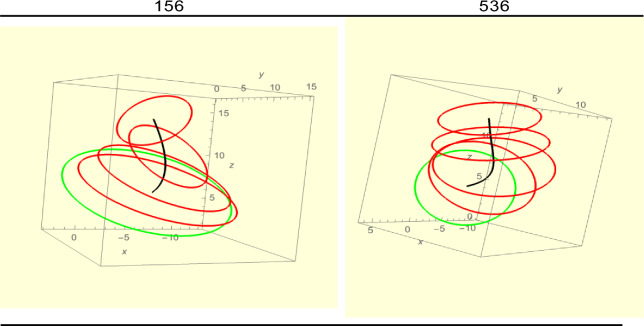
Ear 156 shows greater twist than ear 536.

For ear 536 the maximum $\phi$ and minimum $\psi$ occur on the same *Z*-value, that is, on the *same* slice. But a closer look at ears 536 and 156 shows up differences. See Fig. 6. Only a few slices are shown for greater clarity. Recall that the green slices are at the entrance of the ear (or close by). In both, as the medial line (in black) bends, the slices also rotate. For ear 536 there is a greater turn ($\theta$ motion) which is associated with the normal, and which is approximately the direction of the medial line. The twist of ear 156 is considerably greater.

Starting with ear 156, we can ‘transform it’ to ear 588. The idea behind this is to find ears that might be ‘mirror images’ of each other. First, we reflect 156’s *Y*-axis about the *X*-axis. What this means is that, if with ear 156 we move right of the *X-*axis, in ear 588 we move left of the *X*-axis instead. This step does not change *X*(*Z*) but it reverses the senses of $\phi$ and $\psi$, thereby introducing sign changes in them. If a critical point is added to *X*(*Z*), a compensating critical point must also be added to $\phi$ because of the requirement *T* = *R*. We also see that the twists and writhes of the ears are negatives of each other and now understand why this is so. Note, this analysis does not need the twists and writhes to have the same absolute value. Moreover, the ‘transformations’ never crumple or cut the ears. Finally note that ear 127 has an exceptionally large twist, almost three quarters of a full turn. It is the largest among all the ears analyzed in this paper. For most ears the writhe is small, i.e. $0.1,$ almost negligible. Ear 127 also displays a sudden jump in the tenth slice giving it a ‘stooped’ appearance. Although ears 156 and 536 have small writhe the latter has the larger one. This difference is confirmed by observing the medial line (black) in both. In ear 536 this line shows greater bending and even *coiling.* As noted above ear 156 has the larger twist.

In line with Fig. 4b, we observe for Type-1 ears that when a minimum occurs in the $\theta$ plot, it is also accompanied by a critical point on the *same* slice for the *Y*-plot. Ear 588 in particular shows an almost flat *Y*(*Z*) plot toward the end which is also exhibited by the $\theta$-plot for the same range.

In sum, what we see with Type-1 ears is that the ‘translational’ aspects match well, that is, there is a one-to-one relation with the ‘rotational’ descriptors. A critical point in the former finds a corresponding response in the latter. Of course, the shape of the ear canal is conditioned by the bones and muscles of the head. This coordination exhibits the unity in the geometric and topological aspects of the canal.

### Type-2(L): T = R − 1

**Figure 7:**
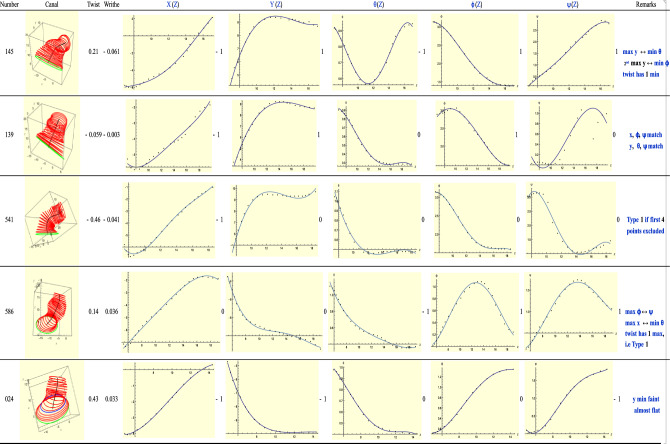
A sample of Type-2 (left) ears. The symbol $\leftrightarrow$ indicates that both critical points occur on the same slice.

Type-2 ears satisfy the condition *T* = *R *− 1. See Fig. 7. For ear 145, its *X*(*Z*) graph has a small minimum at the start. This is matched by an almost imperceptible maximum at the beginning of $\phi$ (follow the dots, not the curve, which is not as accurate).*Y*(*Z*) shows an S-shaped bend between *Z* = 11 and *Z* = 16, and is matched by the minimum of $\theta$ at *Z* = 11 and the maximum of $\psi$ at *Z* = 16. The twisting rate has a minimum so *R* is really 0, i.e. *R* = *T*. We will explain this rationale more fully with Type-3(L) ears below where more examples will be found. It suffices to say that ear 145 is superficially a Type-2 ear but, on closer examination, it is really a Type-1 ear.

Ear 586 has the maxima of $\phi ,\psi$ on the same slice and the maximum of *X*(*Z*) and minimum of $\theta$ on a similar slice. Only the small minimum of *X*(*Z*) close to its beginning is unmatched. Like ear 145 above, ear 586 is of Type-1 if we look at its twist which has index 1.

Ear 586 and ear 541 have small writhes with opposite signs. One notices the coiling of their medial lines in opposite senses. Excluding the first four data points, ear 541 has *T* = 0 so it is really of Type-1. The exclusion of the first four points is because they most likely lie *outside* the canal as suggested by the large axes lengths of their corresponding ellipses.

We still observe the trend noted with Type-1 ears: when a minimum occurs in $\theta ,$ it is also accompanied by a critical point on the *same* slice for the *Y*-plot. However, other trends are also possible. In the case of Ears 139 and 024, if the $\theta$ plot followed a trend as shown in Fig. 4b instead of what is shown here (i.e. as in Fig. 4a), they would be Type-1 ears, as one may verify.

### Type-3(L): T = R + 1

**Figure 8:**
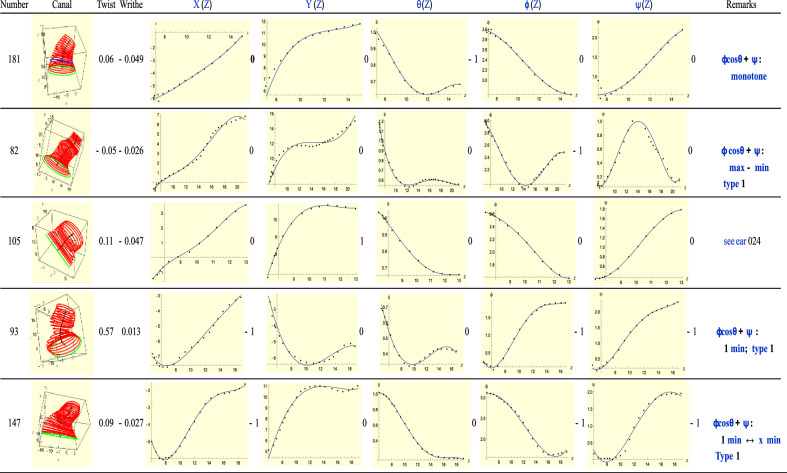
A sample of Type-3 (left) ears.

For Type-3 ears *T* = *R* + 1 holds. See Fig. 8. For this type, the twisting rate $\phi cos\theta +\psi$ (see Eq. (4), and called *twist* for short hereon) on the plane of the slice, is helpful. In using this *twist*, we are generally able to reduce the number of critical points of the ‘rotational’ group. (However, due to space constraints we have not displayed the twist graphs.) We can see this with the second ear of Fig. 8, namely 82 and most of the other ears. For ear 82, the total twist is 0 due to cancellation of maximum and minimum contributions so index *R* = 0. Thus we have, *T* = *R*, which is of Type-1. For ear 82 the green ellipse is significantly larger than the others so it is likely that this is a slice *outside* the canal. Alternatively, it may happen that the critical points of two graphs of the ‘rotational’ type fall on the same point, which means they count as only one critical point. In this way we are able to reduce them to Type-1 again as in the case of ears 147 and 93. Ear 105 is very similar to ear 024 (Type-2) which we mentioned above: instead of a minimum in *y*[*Z*] for the later, we have a maximum for the former.

For ear 181, we find that the minimum of the $\theta$ graph is unmatched by the translation plots. Between *Z* = 11 and *Z* = 15, *Y*(*Z*) advanced by about 1 mm while $\theta$ stayed very of close to $\theta \approx 0.6 \mathrm{rad}.$ We had anticipated this situation in Fig. 4.

### Type-4(L): T = R − 2

**Figure 9:**
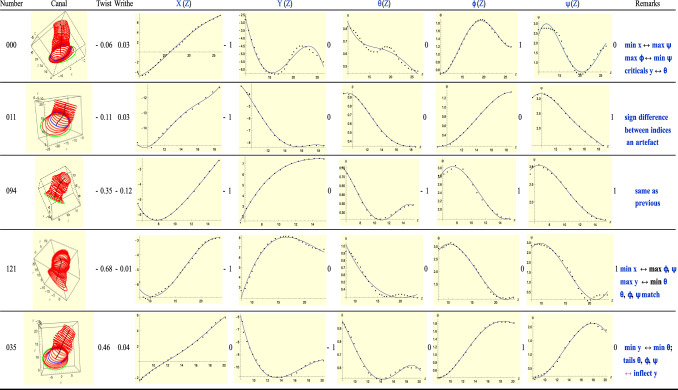
A sample of Type 4 (left) ears.

Type-4 ears satisfy the relation *T* = *R *− 2. See Fig. 9. For the first, ear 000, the minimum of *X*(*Z*) and maximum of $\theta$ match as do the minimum of *Y*(*Z*) and minimum of $\theta$. The critical points of *Y*(*Z*) and $\theta$ occur on the same slices. Similar observations can be made of the next ear, 011. That the indices of the *T* and *R* critical points may have opposite sign, at times, is only an artefact. For instance for ear 011, the minimum for *X* occurs for negative values of *X*. But that region of *X*(*Z*) corresponds to large (or maximum) values of angle $\psi$. Thus, there is no contradiction between the signs of the critical points of *X*(*Z*) and $\psi \left(Z\right)$. For ear 094, the critical points of *X*(*Z*) and $\phi \left(Z\right)$ occur on the same slice and ‘cancel’ each other. As with ear 000, for ear 094 the sign difference between critical points of *X*(*Z*) and $\psi \left(Z\right)$ is again an artefact, as with ear 011. The critical points of $\theta$ and $\psi$ also ‘cancel’ each other but they do not occur on the same slice. Similar remarks apply to ear 035.

We continue observing the trend we saw with Type-1 when a minimum occurs in $\theta$ it is also accompanied by a critical point on the *same* slice for the *Y*-plot. See ears 000, 121, 035.

### Type-5(L): T = R + 2

**Figure 10:**
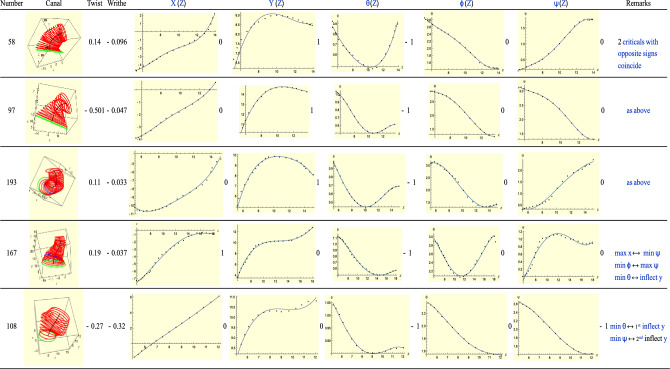
A sample of Type 5 (left) ears.

Type-5 ears correspond to *T* = *R* + 2. See Fig. 10. The first three share similar features: they have a maximum for *Y*(*Z*) and a minimum for $\theta$. These critical points occur at exactly the same slice. This feature is characteristic of a ‘*monkey* saddle’ surface for which a point is a maximum in one direction and simultaneously a minimum in a perpendicular direction. In fact this ‘coincidence’ also occurs for the next ear, ‘167’ and, indeed, for the remaining two ears of Fig. 9. Ear 108 displays both a large twist and large writhe (-0.32, the largest in our collection). Its *Y*(*Z*) shows two portions that are almost flat (follow the points *not* the curve which misses the data points) and that coincide with the minima of $\theta$ and $\psi$. For *Y*(*Z*) these portions correspond to points of inflection, i.e. the second derivative of *Y*(*Z*) vanishes. A finer resolution of the point cloud would give more information about these points.

For the writhe *not* to be small (> 0.15), which is rare in this study, three conditions must occur simultaneously: (a) both $\phi$ and $\psi$ must vary across a large angular range, greater than $1$ rad in the *same* sense; (b) $\theta$ should not be close to $\theta =0$; and (c) $\theta$ should vary slowly compared with $\phi$ and $\psi$. As we can see, these three conditions hardly coincide for most ears. But they occur for ear 108.

### Types-6 & 7 (L)

The last two ear Types are Type-6 and Type-7, characterized by *T* = *R*—3, *T* = *R* + 3, respectively. Left and Right ears combined, Type-6 and Type-7 comprise only 9% of the total collection of ears.

**Figure 11:**
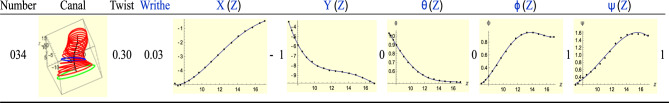
Type 6 left ear 034. The green ellipse has large axes which might be due to its being outside the canal.

Figure 11 shows Ear 034, a Type-6 left ear for which *T* = *R* – 3. The only translational critical point occurs in *X*(*Z*). But it is likely that this minimum is *outside* the ear canal, which means it does not count. Rotationally, both $\phi$ and $\psi$ exhibit single maximum points. The total length of the canal that is examined in this case is 9-mm long. It appears that the rotational critical points vanish for canal lengths 1 mm longer because both $\phi$ and $\psi$ graphs could rise upward yielding a total index of *R* = 0. This possibility is supported by (*X*(*Z*), *Y*(*Z*)) lying in the third quadrant of the *XY* plane and we expect $\psi$ to grow toward the end of its trajectory.

**Figure 12:**
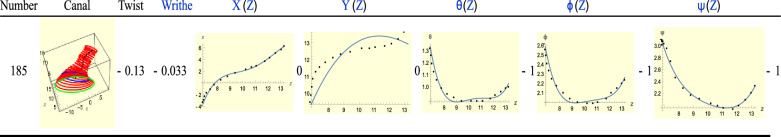
Type 7 left ear 185. Notice the ear contracting toward the end.

Ear 185, shown in Fig. 12, is an example of a Type-7 left ear satisfying *T* = *R* + 3. Both *X*(*Z*) and *Y*(*Z*) are monotonic so *T* = 0. All three rotational graphs display one minimum each, hence *R* = − 3. These occur in slices close to each other and indeed the medial line does curve in that region. The angular ranges are smaller than most ears we have seen. The canal is more constricted as well in that part of the ear, toward the end. We might infer that as the ear canal becomes constricted, the ‘coordination’ between translational and rotational degrees is less clear.

**Figure 13:**
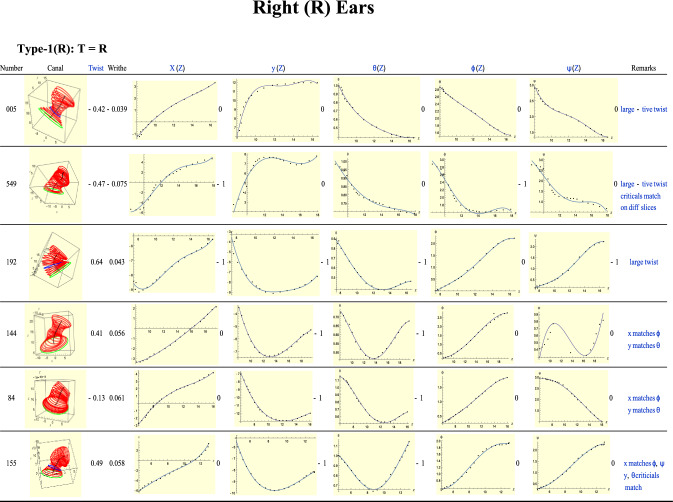
A sample of Type-1 (right) ears.

Figure 13 displays some representative Type-1 (right) ears for which *T* = *R*, as with the left ears. The first, ear 005, has no critical points. Follow the dots rather than the fitting curves. All graphs are monotonic. Because the rotations $\phi ,\psi$ have the same sense, the twist is large and negative. For the second ear, 549, similar remarks apply. But now there are critical points in the *X, Y* and $\phi$ plots. Although the critical points match, they do not occur in the same slices. The next ear, 192, exhibits a large positive twist, almost two-thirds of a turn. Interestingly, the critical points of *x*(*Z*) and *y*(*Z*) match those of $\theta \left(Z\right)$ and $\psi (Z)$.

The last three ears 144, 84 and 155, can be discussed together. All have very similar graphs except for their $\psi$ plots. In fact, all three display quite different behaviour in their $\psi$ plots. Their *X* plots are monotonic and rising. All three ears show a critical point in their *Y* plots, which are also matched by their $\theta$ plots, which in turn look similar. Ear 84 shows a negative twist compared to the large and positive twist of the other two. It is easy to see that this is due to the fact that for ear 84 the $\phi ,\psi$ plots turn in opposite senses. It is also easy to understand why the twist of ear 155 is larger than that of ear 144. We had seen these same features with Type-1 left ears.

In Fig. 5 we saw two Type-1 left ears, 156 and 588, which resembled mirror reflections of each other. Here in Fig. 13, ears 549 and 155 bear a similar relation with each other.

None of the ears exhibits a notable writhe. In fact this can be said about all the right ears discussed in this work. We arrive at the conclusion that, like the Type-1 *left* ears, these Type-1 *right* ears also display the same coordination that we had seen in the former.

### Type-2(R): T = R − 1

**Figure 14:**
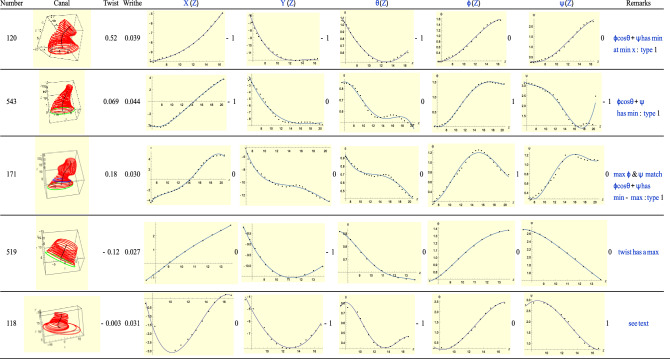
A sample of Type-2 (right) ears.

Type-2 ears correspond to *T* = *R *− 1. See Fig. 14. For ear 171, the critical points of $\phi ,\psi$ occur on the same slice. Moreover, the total twist $\phi \mathrm{cos}\theta +\psi$ has a maximum and minimum, i.e., it has index 0. This makes ear 171 a Type-1 ear. This observation may also be verified for a number of other ears in Fig. 14. Both ears 120 and 171 have larger twist than the rest. For both the $\phi$ and $\psi$ are in the same sense but for ear 171 these rotations are not monotonic unlike those for ear 120. This explains why ear 120 has the much larger twist.

For ear 118, *Y*(*Z*) and $\theta$ minima are found on the same slice. The minimum of the *X-*plot corresponds to a point on the third quadrant of the *XY* plane and has a value of $\psi$ which consistent with what the $\psi$ plot shows. However, a small maximum toward the end of *X*-plot is unmatched by any rotation graph. For ear 519, all graphs are monotonic except the *Y-*plot which shows a single minimum which is not matched by any of the rotational plots.

For Ear 519 the twist has a maximum. This means that *T* = − 1 while *R* =  + 1. However, the difference in signs can be explained if we observe that *XY* plot lies in the third and fourth quadrants. As with Type-4 (L) graphs this feature is an artefact and implies no inconsistency.

### Type-3(R): T = R + 1

**Figure 15:**
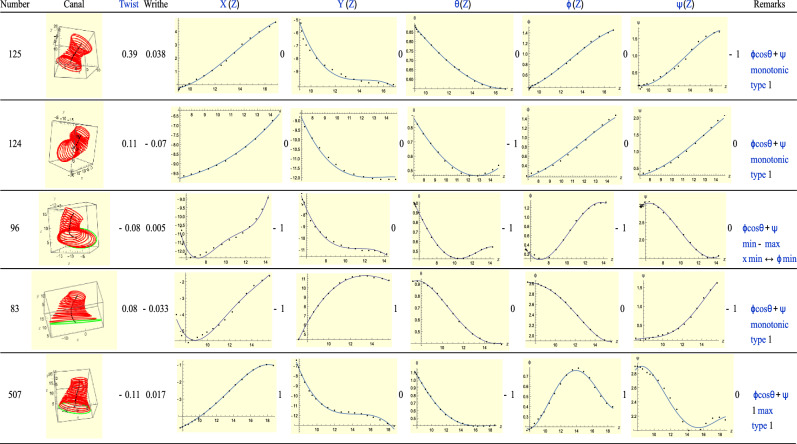
A sample of Type-3 (right) ears.

Type-3 ears correspond to *T* = *R* + 1. See Fig. 15. As was the case with Type-3 (left) ears, here the total twist $\phi \mathrm{cos}\theta +\psi$ assists us to reduce the index of some of the rotational graphs. In fact we see this in all the ears of Fig. 15. The critical points do not always fall on the same slice, as we usually see in Type-1 ears. We see also the same phenomenon of large and small twist. For ears 124 and 507 the phenomenon of Fig. 4 is clearly illustrated by the *Y* and $\theta$ plots. Thus Type-3 ears, both left and right, share much in common.

Closer examination of ear 125 reveals that the only critical point occurs at the beginning of the $\psi$ plot. This may be an artefact in that we are at the entrance of the canal, but we could well be *outside* the canal. This has happened in a number of instances, for instance, to ear 83 in Type-3 left ears. See remarks accompanying it. For future studies it will be helpful to adopt a uniform scanning method that only takes the point cloud data from the canal, not outside it.

### Type-4(R): T = R − 2

**Figure 16:**
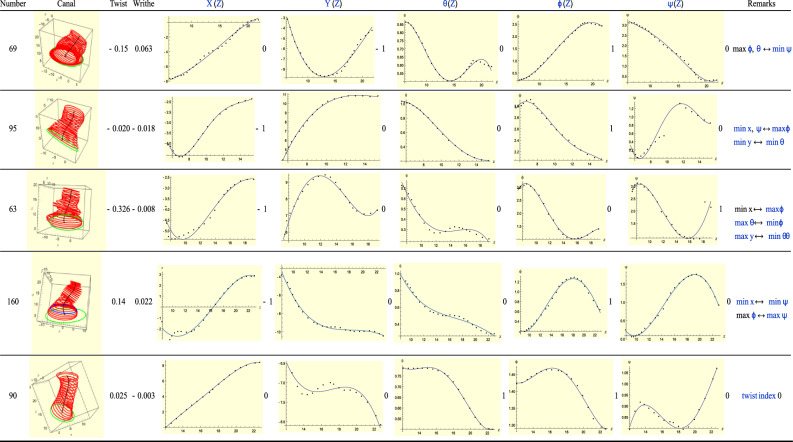
A sample of Type-4 (right) ears.

Type-4 ears correspond to *T* = *R *− 2. See Fig. 16. For Ear 95 the *X–Y* motion is in the second quadrant and the opposite trends of *X*(*Z*) and $\phi$ is implied by this observation. Similarly, for Ear 63, but this time it is *X*(*Z*) and $\psi$ that are paired. For these two ears, the fact that *T* and *R* have opposite signs is because of the same reason we gave for Type-4 (L) ears: this sign difference is really an artefact of our coordinate system. For ear 160 several critical points once again occur very close to each other. The ones that matter are the minima of *X* and $\psi$, which are on the same slice. The twist has a minimum and maximum, i.e. it has index 0. Effectively then the rotational graphs have *R* = 0. The *T* = − 1 critical point is matched by the minimum of $\psi$. Note, however, that the minimum of *X* at $Z\approx 16$ looks like a point of inflection, effectively reducing *T* to 0. This suggests that ear 160 is really a Type-1 ear. Note also that the critical point of *X*(*Z*) might well be outside the canal, in which case it does not count, another reason why we could expect *T* to equal zero.

Finally ear 90 is interesting because *T* = 0 while *R* =  + 2. The total twist, however, has a maximum and minimum, i.e. its index is 0. This implies that *R* =  + 1 because of the almost imperceptible maximum of the $\theta$ graph. This maximum is almost a point of inflection and is accompanied by the almost ‘flat’ *Y*-graph, where the graph shows small ‘oscillations.’ Ear 90 below, along with ear 139 in Fig. 7, have the smallest writhe and indeed both resemble a featureless 1D pole.

For Ear 69 the *XY* motion is in the lower half plane and the combination of $\phi$ and $\psi$ plots is consistent with this.

### Type-5(R): T = R + 2

**Figure 17:**
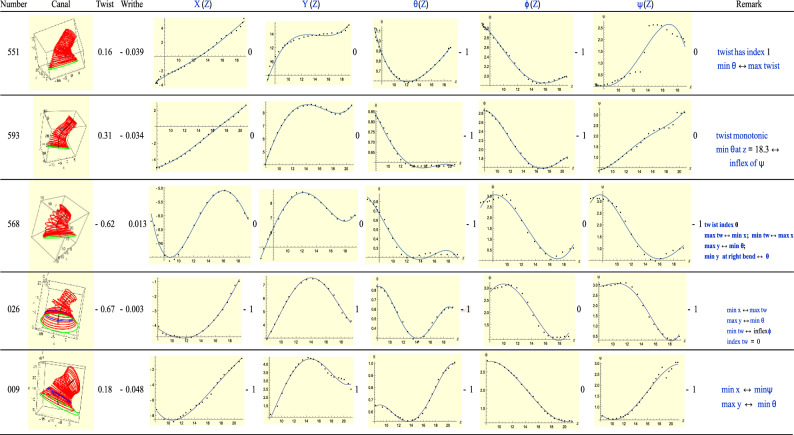
A sample of Type-5 (right) ears.

Type-5 ears correspond to *T* = *R* + 2. See Fig. 17. Ear 551 has *T* = 0 and *R* = − 2. The total twist has a maximum, i.e. its index is + 1. Along with the index of the $\theta$-plot, the effective value of *R* is 0, consistent with *T*. For the next ear, 593, its twist is monotonic, i.e. its index is 0. The minimum of $\theta$ toward the end and $\psi$ in the same region parallel the behavior of *Y*(*Z*) in the same range. That is, this situation follows the line of Fig. 4. This analysis applies to the next two ears.

Ear 009 has two critical points among the *X*(*Z*) and *Y*(*Z*) graphs and another pair among the $\theta ,\psi$ graphs. The *X* critical point goes with the minimum of $\psi$ while the maximum of *Y* with the minimum of $\theta$. The latter pair have opposite signs (a maximum and a minimum) and they occur on the same slice. This is again an example of a monkey saddle.

For ear 026 the critical points of the *X* and *Y* plots are matched by those of the rotational degrees of freedom. Toward the end of the canal there is very slight turning in $\phi$ and $\psi$ which has a minimum. The maximum of *Y*(*Z*) and minimum of $\theta$ remind us Fig. 4 once again.

**Figure 18:**
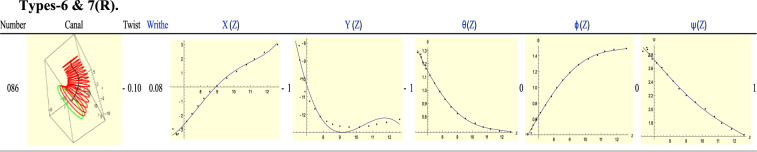
Type-6 right ear 086.

Figure 18 shows ear 086, a Type-6 right ear. For this ear, *T* = -2 while *R* =  + 1 so *T* = *R* – 3. The first minimum, which is at the beginning of *X*(Z) is matched by a maximum at the same slice by $\psi \left(Z\right).$ As with Type-4 ears, the sign difference of the indices is an artefact of our convention. Only the minimum of *Y*(*Z*) is unmatched. In fact this behaviour had been anticipated in Fig. 4 if we consider the slow decay of the $\theta$-plot.

**Figure 19:**
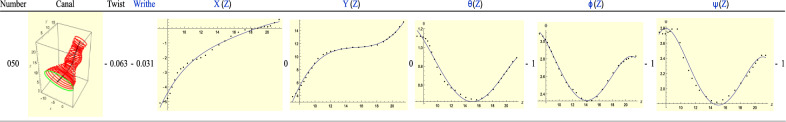
Type-7 right ear 050.

Finally Fig. 19 displays ear 050, a Type-7 right ear satisfying *T* = *R* + 3. Total twist and writhe are small. Both translational graphs are monotonic. Each rotational graph has a single minimum. Closer examination shows that all three minima fall within one slice of each other. Also *Y*(*Z*) shows a flat section in the same region, while *X*(*Z*) has a constant upward slope, which is consistent with decreasing $\phi$ and $\psi$. These angles increase when the flat portion of *Y* is replaced by a rising *Y*. What the minima are telling us is that the rotations *past* the minima are being retraced but backwards. This is a kind of reversibility in motion.

## Discussion

From the data collected, can we find some distinguishing features peculiar to ears of one type or of a given side of the head? A first attempt might be to compare the indices of the *X, Y* and $\theta$ degrees of freedom (d.o.f.), as shown in Tables 1 and 2. These indices are + 1, 0, − 1, depending on whether the plots have a net maximum, are monotonic or have a net minimum. Note that it is impossible, for instance, to have two maxima, unless there is one minimum between them. Table 1 displays the normalized numbers of ears having these indices for the left ears. The corresponding numbers for right ears are displayed in Table 2. Comparing the entries with these indices for their *X, Y*, $\theta$ plots, it is difficult to tell which is a left ear and which is a right.

**Table 1:** Normalized distribution of indices for *X, Y* and $\theta$ plots for left ears.

d.o.f	*X*			*Y*			$\theta$		
Type	1	0	− 1	1	0	− 1	1	0	− 1
1	0	9.6	17.3	3.8	16.3	6.7	0.9	9.6	16.3
2	0	6.7	8.6	1.9	10.6	2.9	0.9	6.7	7.7
3	0	11.5	10.5	3.8	18.2	0	0	5.7	16.3
4	11.5	5.7	0	1	10.6	5.7	0.9	1.5	3.8
5	1.9	8.6	0	2.8	7.7	0	0	0	10.6
6	0	0	2.9	0	1.9	1	0	2.9	0
7	0	4.8	0	1.9	2.9	0	0	0	4.8
Total (%)	13.4	46.9	39.3	15.2	68.2	16.3	2.7	37.7	59.5

**Table 2:** Normalized distribution of indices for *X, Y* and $\theta$ plots for right ears.

d.o.f	*X*			*Y*			$\theta$		
Type	1	0	− 1	1	0	− 1	1	0	− 1
1	0	14.3	5	1.7	9.2	8.4	0.8	9.2	9.2
2	0.8	9.2	11.7	0	15.1	6.7	0	10.9	10.9
3	0.8	18.5	10.9	5.9	24.3	0	0	5	25.2
4	0	1.7	5	0	5	1.7	0.8	5.9	0
5	0	6.7	3.3	0	6.7	3.3	0	0	10.1
6	0	0	2.5	0	0	2.5	0.8	1.7	0
7	3.3	5.2	0.8	3.4	5.9	0	0	0	9.2
Total (%)	4.9	55.6	39.2	11	66.2	22.6	2.4	32.7	64.6

The number of 0 indices ears is a standout feature of the dataset: in general, the *X* and *Y* plots with 0 index outnumber the rest. These plots are monotonic. Then there are a good number that exhibit a minimum, i.e., index -1. But very few show a maximum, i.e., index + 1. The notable exception is Type-4 left ears which show a maximum more often, but there are relatively few Type-4 ears in the dataset (see Table 3). Types-5 and 7 for both left and right ears overwhelmingly display a minimum $\theta$. For Type-3 ears we see the right ears strongly tending to display a minimum $\theta$ while the left ears exhibit this tendency but less strongly. None of these observations, however, gives us a strong indication of a feature unique to one type of ear or a side of the head.

**Table 3:** Classification Types of ear canals along with percentages of ears for each type.

Type	Description	% Left ears	% Right ears
1	*T* = *R*	27	19
2	*T* = *R *− 1	15	22
3	*T* = *R* + 1	22	30
4	*T* = *R *− 2	17	7
5	*T* = *R* + 2	10	10
6	*T* = *R *− 3	3	2
7	*T* = *R* + 3	6	10

Lacking a distinguishing feature, we might instead search for a unifying element. Recall the last paragraph of the section on Type-1 left ears: *the translational aspects* (*T*) *match well, that is, are in one-to-one relation, with the rotational* (*R*). This observation holds good with Type-1 *right* ears as well. Type-1 ears satisfy *T* = *R*. From this point, we consider Type-2 ears, for which *T* and *R* differ by − 1. When we carefully examine Figs. 7 and 14 and their accompanying remarks, we see that the translational and rotational aspects generally agree. However, in the case of Type-2 (left or right) ears this agreement becomes more explicit when we calculate the total twist (5). This is because the Euler angles $\phi$ and $\psi$, which combine to yield total twist, are just kinematic operations that rotate the elliptic plane about the ${Z}{\prime}$-axis and the z-axis, respectively (see Fig. 2b). The total twist describes the net rotation of the elliptic plane. Just to give more emphasis to this observation, we can verify that for Type-1 ears the total twist leaves the defining relation *T* = *R unchanged*. Therefore, when we introduce the generalized relation for total twist, the difference of -1 between *T* and *R* for Type-2 ears is spurious. In this sense Type-2 ears are truly also Type-1 ears. The coordination between translations and rotations continues to hold for Type-2 ears.

When we continue this analysis with Type-3 ears, which satisfy *T* = *R* + 1 (see Figs. 8, 15), we find once again that total twist reduces Type-3 ears into Type-1 ears and the conclusion from the preceding paragraph continues to hold here.

With Type-4 ears, which satisfy *T* = *R *− 2, a new trend emerges. When we examine the first three (left) ears of Fig. 9, we see that the defining relation for this type arises because a minimum in the *X*-plot corresponds to a maximum in *ϕ* or *ψ*. When we carefully examine the definition of these angles (see Fig. 2b), we see that they are large and are in the region where *X* is negative. Therefore, the sign difference, where *X* has a minimum while the *ϕ* or *ψ* angles have a maximum, is simply a result of our sign conventions and is not an intrinsic distinction. For Type-4 *right* ears we observe that Ears 95 and 63 of Fig. 16 are similar to the first three ears of Fig. 9. Thus, the correspondence between *T* and *R* continues to hold.

For Type-5 left ears, which satisfy *T* = *R* + 2, we see for the first three ears of Fig. 10 that it is the maximum of *Y* that is now matched with the minimum of $\theta$. This relationship is similar to the case with Type-4 ears. For Type-5 *right* ears the remarks connected with Fig. 17 show that ear 551 is a Type-1 ear while for ear 593 it is the $\theta$ and $\psi$ plots that could provide the required correspondence. Once again, as with Type-4 ears, we find consistency.

The descriptions of Type-6 and Type-7 ears didn’t provide a clear picture, but suggested coordination between the T and R degrees of freedom. Further research on these ears should clarify matters. It’s reassuring that Types-6 and 7 ears make up only about 9% of the total number of ears.

## Conclusions

We analysed 300 ear-canal shapes by extracting descriptors from the way the ear canal translates, rotates, and writhes. After removing shapes with scan defects or other inconsistencies, 250 point cloud ear canals remained for analysis. Processing of this data gave us the shape of the medial line running through the centers of the ellipses, and the orientation of the slices along the canal length. The twist and writhe of each ear were calculated.

We classified each ear by comparing the critical points of the medial line with the corresponding critical points of the Euler angles that describe each elliptic slice. The sum of the indices of the medial line was termed the translational index, *T*, while the sum of indices of the Euler angles was called the rotational index, *R*. Our classification compared the difference between *T* and *R* as shown in Table 3.

We aimed to identify and compare ears of different types and find relationships between the types. To do this classification the relationship between T and R was analysed, which revealed seven different ear types. Implicit in this classification is the possible correlation between canal shape and the ear types. The last two types, 6 and 7, comprised only 9% of the ears analysed. By far, Types 1, 2, 3 were the more numerous with substantial numbers of Types 4 and 5.

Studying the medial line of the scanned ear canals we established geometrical connections that could the reveal reasons for some ears having larger twists than others. In a very few cases we saw large writhes and traced their origins. The concepts of twist and writhe could be useful in efforts to calculate the energy that can be harvested from ear-canal movements^23^. This may have downstream implications to hearing aid rehabilitation. Although real-ear measurement (REM) is still the gold-standard for hearing aid verification, it may not be accessible to all. Since the acoustic properties of the ear are affected by the ear canal shape^24^, it is reasonable to suggest that studying the resonant modes within the ear canal may be used to detect the seven ear-canal types. The present study lays the groundwork for predicting ear canal shapes from incomplete scans, such as those obtained by directly scanning ears with a structured light scanner^25^. Moreover, it may also be used to model an ear acoustic response and consequently the amplification targets that need to be matched by hearing aids. Ear canal classification may also help with accurate placement of the receivers and shaping of the ear molds to optimize amplification and comfort of fit.

## Supplementary Information

Supplementary Information.

## Data Availability

All data analysed in this study is available from the corresponding author on reasonable request. In the Supplementary Information we provide data for the first 100 ears.
