# Supplementary material for: Lexicon for classifying ear-canal shapes
Source: Sci Rep. 2023 Jul 22;13:11866. doi: 10.1038/s41598-023-38570-3 (PMC10363131; doi:10.1038/s41598-023-38570-3)
Supplement: Supplementary file 1 — Supplementary Information. [file 41598_2023_38570_MOESM1_ESM.zip › Supplementary/Supplementary Information.docx]

***Supplementary Information***

**to accompany**

**Lexicon for classifying ear-canal shapes**

J. C. Martinez*^1^, Goh Zhi Hwee^1^, Luis Yap^2^, Kenneth Wei De Chua^3^, Savitha Kamath^3^, Conrad Kang Rui Chung^3^, Wendy Yu Bing Teo^3^, Charmaine Kai Ling Tan^1^, Stylianos Dritsas^1^, Robert E. Simpson^4^

^1^*Singapore University of Technology and Design (SUTD), 8 Somapah Road, 487372, Singapore*

^2^*University of Sydney, New South Wales, 2006, Australia*

^3^*Changi General Hospital, Department of Otorhinolaryngology-Head and Neck Surgery, Singapore*

*^4^School of Engineering, University of Birmingham, Birmingham B15 2TT, UK*

Our data for about 100 ears is provided in the following Excel files (*Ears data*). Each file, numbered consecutively from 0000 onwards, contains the data for the elliptic slices of each ear, that is, a row in the Excel data file corresponds to data pertaining to a single elliptic slice as described in the Method’s section of the article. Each ear is comprised by 12 to 36 slices.

Specifically, for every slice the data consists of (a) the coordinates of its center (*X*_0_*, Y*_0_*, Z*_0_) [these are the first three columns]; (b) the length of the semi-major axis [fourth column]; (c) the directions (or direction cosines) of the semi-major axis [the fifth to the seventh columns]; (d) the length of the semi-minor axis [the eight column]; (e) the directions of the semi-minor axes [ninth to the eleventh columns]. See Fig. 2(b) of the article.

Another Excel file (*Ears statistics*) provides the following information for the same ears: whether the ear is on the right or left; the volume, area and length of the ear canal. All linear units are in mm.
